# Personalising genetic counselling (POETIC) trial: Protocol for a hybrid type II effectiveness-implementation randomised clinical trial of a patient screening tool to improve patient empowerment after cancer genetic counselling

**DOI:** 10.1186/s13063-023-07723-0

**Published:** 2023-11-08

**Authors:** Laura E. Forrest, Erin Tutty, Anurika P. De Silva, Lara Petelin, Amy Ruscigno, Rebecca Purvis, Katrina Monohan, Maira Kentwell, Adrienne Sexton, Lesley Stafford, Paul A. James

**Affiliations:** 1 Parkville Familial Cancer Centre, Peter MacCallum Cancer Centre, Locked Bag 1, A’Beckett St, Melbourne, Victoria 3008 Australia; 2 Parkville Familial Cancer Centre & Genomic Medicine, Royal Melbourne Hospital, Parkville, Victoria Australia; 3 Sir Peter MacCallum Department of Oncology, The University of Melbourne, Melbourne, Victoria Australia; 4 Department of Medicine, The University of Melbourne, Melbourne, Victoria Australia; 5 Centre for Epidemiology and Biostatistics, Melbourne School of Population and Global Health, The University of Melbourne, Melbourne, Victoria Australia; 6 Methods and Implementation Support for Clinical and Health (MISCH) research Hub, Faculty of Medicine, Dentistry and Health Sciences, The University of Melbourne, Melbourne, Australia; 7 The Daffodil Centre, The University of Sydney, a joint venture with Cancer Council New South Wales, Camperdown, Australia; 8 Melbourne School of Psychological Sciences, The University of Melbourne, Melbourne, Victoria Australia

**Keywords:** Genetic counselling, Hybrid effectiveness-implementation trial, Patient screening tool, Randomised control trial

## Abstract

**Background:**

Genetic counselling aims to identify, and address, patient needs while facilitating informed decision-making about genetic testing and promoting empowerment and adaptation to genetic information. Increasing demand for cancer genetic testing and genetic counsellor workforce capacity limitations may impact the quality of genetic counselling provided. The use of a validated genetic-specific screening tool, the Genetic Psychosocial Risk Instrument (GPRI), may facilitate patient-centred genetic counselling. The aim of this study is to assess the effectiveness and implementation of using the GPRI in improving patient outcomes after genetic counselling and testing for an inherited cancer predisposition.

**Methods:**

The PersOnalising gEneTIc Counselling (POETIC) trial is a hybrid type 2 effectiveness-implementation trial using a randomised control trial to assess the effectiveness of the GPRI in improving patient empowerment (primary outcome), while also assessing implementation from the perspective of clinicians and the healthcare service. Patients referred for a cancer risk assessment to the conjoint clinical genetics service of two metropolitan hospitals in Victoria, Australia, who meet the eligibility criteria and consent to POETIC will be randomised to the usual care or intervention group. Those in the intervention group will complete the GPRI prior to their appointment with the screening results available for the clinicians’ use during the appointment. Appointment audio recordings, clinician-reported information about the appointment, patient-reported outcome measures, and clinical data will be used to examine the effectiveness of using the GPRI. Appointment audio recordings, health economic information, and structured interviews will be used to examine the implementation of the GPRI.

**Discussion:**

The POETIC trial takes a pragmatic approach by deploying the GPRI as an intervention in the routine clinical practice of a cancer-specific clinical genetics service that is staffed by a multidisciplinary team of genetics and oncology clinicians. Therefore, the effectiveness and implementation evidence generated from this real-world health service setting aims to optimise the relevance of the outcomes of this trial to the practice of genetic counselling while enhancing the operationalisation of the screening tool in routine practice.

**Trial registration:**

Australian New Zealand Clinical Trials Registry registration number 12621001582842p. Date of registration: 19th November 2021.

**Supplementary Information:**

The online version contains supplementary material available at 10.1186/s13063-023-07723-0.

## Background

Genetic counselling and testing for cancer predisposition syndromes are increasingly being offered to individuals with and without cancer diagnoses due to the evidence of improved health outcomes for those identified with a germline pathogenic variant (PV) [1, 2]. Individuals without cancer who are aware they have inherited a cancer-causing germline PV have the opportunity to engage in efficacious cancer risk management strategies demonstrated to reduce cancer-related morbidity and mortality [3, 4]. For those with cancer, the diagnosis of a germline variant offers treatment options [5, 6], refines prognosis, and defines future cancer risks with associated risk management strategies. The genetic counselling process facilitates informed decision-making about genetic testing and any subsequent risk management options [7], and promotes patient empowerment and adaption to genetic status after receipt of results [8, 9].

Gold standard genetic counselling is patient-centred, with patient needs identified and addressed by skilled practitioners. The genetic counselling process facilitates informed decision-making about genetic testing, supports empowerment and adaptation to genetic information, and promotes communication of genetic information to at-risk family members [8, 10–13]. The provision of genetic counselling by the multidisciplinary teams staffing cancer-specific clinical genetics services (Familial Cancer Centres), increasing demand upon the limited genetic counsellor workforce [14], and the increasing provision of genetic testing by medical practitioners who work externally to these clinical genetics services mean that the provision of genetic counselling may vary in quality and quantity [15]. Therefore, transformative approaches to genetic counselling practice are required to ensure consistent, patient-centred care prevails [16, 17].

Screening tools, often used in other healthcare settings to efficiently identify patient needs, may facilitate genetic counselling and support patient-centred care. Deployment of patient screening tools in clinical oncology settings is becoming routine, yet examples of routine use in clinical genetics services have not been published. This is despite the existence of specifically developed and validated psychosocial screening tools for use in clinical genetics [18, 19]. The Genetic Psychosocial Risk Instrument (GPRI) is a validated genetics-specific screening tool designed to identify psychological risk factors that predict distress in patients undergoing genetic testing [19].

The GPRI was piloted with patients and clinicians (genetic counsellors, clinical geneticists, and medical oncologists) in the Parkville Familial Cancer Centre (PFCC), in Victoria, Australia, from December 2018 to March 2019 and it was determined that this screening tool was acceptable and feasible to patients and clinicians, and useful for clinicians during appointments [20]. While the rigorous development and validation of the GPRI together with the demonstrated acceptability, feasibility, and usefulness make this an ideal genetic counselling intervention, the GPRI has not been evaluated regarding its effectiveness in improving patient outcomes or whether it can be implemented routinely into practice. Therefore, the objective of this study is to test the use of the GPRI and examine the implementation of this screening tool in a clinical genetics service providing cancer genetic counselling.

## Methods

This study is sponsored by the Peter MacCallum Cancer Centre and was approved by the Peter MacCallum Human Research Ethics Committee (HREC/78093/PMCC-2021) on 22 December 2021. Site governance authorisation was granted on 7 February 2022 for the Peter MacCallum Cancer Centre and on 7 October 2022 for the Royal Melbourne Hospital, with a Clinical Trial Research Agreement executed between the sponsor and Melbourne Health. Trial oversight is undertaken by a steering committee comprised of the investigator team (authors LEF, ADP, LP, RP, AS, LS, and PAJ) and two consumer representatives and meets six monthly. The principal investigator (LEF) is responsible for leading the trial, including monitoring enrolment, training of study staff, and providing education sessions to clinicians prior to signing the delegation log. Data management and quality is monitored by co-investigator and biostatistician (ADP), which is presented at the six monthly steering committee meetings. Further, the forced choice and required field options in the REDCap database are used throughout to minimise missing data or invalid responses.

### Research aims

This hybrid type II effectiveness-implementation randomised clinical trial includes two aims:

1. Assess the effectiveness of using the GPRI, relative to usual care, in improving patient outcomes including empowerment (primary outcome) after genetic counselling and testing for an inherited cancer predisposition; and
2. Assess the implementation of the GPRI in genetic counselling appointments in terms of adoption, cost-effectiveness, fidelity, feasibility of routine use, penetration, and sustainability.

### Setting

This research will be conducted at the PFCC, the conjoint clinical genetics service at the Peter MacCallum Cancer Centre and the Royal Melbourne Hospital, located in Victoria, Australia. Patients are referred to the PFCC for a risk assessment based on personal and/or family history of cancer. Referrals are actioned by an intake assistant, with genetic counsellor oversight, who contacts the patient to obtain additional family history and/or clinical information. Once the intake process is complete, an appointment is booked with up to 6 weeks wait for the appointment date. Patients who are deemed at increased risk of an inherited predisposition to cancer and are likely to be offered genetic testing will typically attend two appointments: (1) a pre-test appointment during which consent for genetic testing is discussed and obtained; and (2) a results appointment at which the genetic test result is disclosed. The clinic is staffed by clinicians, comprising genetic counsellors, clinical geneticists, medical oncologists, gastroenterologists, and clinical genetics fellows.

### Study design

A hybrid type 2 effectiveness-implementation trial has been designed to assess the effectiveness of using the GPRI in PFCC appointments while also assessing the implementation strategy (Table 1) [21].

**Table 1:** Hybrid effectiveness-implementation trial map

	**Aims**	**Framework**	**Constructs**	**Outcome**	**Analysis level**	**Measurement**	**Data collection**
**Effectiveness**	To assess the effectiveness of using the GPRI in genetic counselling	FOCUS-GC	Communication strategy	Identify patient psychosocial needs during appointment	Clinician	No. of patient needs identified	Appt. checklist, audio recording
				Address patient psychosocial needs during appointment	Clinician	Y/N patient needs address	
			Process measures	Appointment length	Service	Time (minutes)	Appt. checklist, audio recording
				Referrals for support	Clinician	No. & type of referrals offered	Appt. audio recording
						No. & type of referrals made	Clinical data
				Letters to at-risk relatives	Clinician	No. letters written	Clinical data
			Patient care experiences	Satisfaction with appointment	Patient	Genetic Counselling Satisfaction Scale	t3 & t6
			Patient changes	Empowerment	Patient	Genetic counselling Outcome Scale	−t1, t3 & t6
			Patient health	Distress	Patient	Multidimensional Impact of Cancer Assessment Questionnaire	t6
				Adaption	Patient	Psychological Adaption Scale	t6 & t7
				Cancer risk management	Patient	Uptake of strategies	t7
			Family changes	Communication to at-risk relatives	Patient	No. of family members informed	t7
**Implementation**	To assess the implementation of the GPRI in genetic counselling	Implementation Outcomes Framework	Acceptability	Acceptable to implement GPRI in genetic counselling appointments.	Patient	Acceptability patient survey	Complete
					Clinician	Acceptability staff survey	
			Adoption	Uptake by patient	Patient	GPRI pilot (early stage)	Complete (uptake 94%)
				Uptake by clinician	Clinician	Use by clinician (Y/N)	Appt. audio recording
			Feasibility	Feasible to implement GPRI in genetic counselling	Patient	Feasibility patient survey	Complete
					Clinician	Feasibility staff survey	Complete
						Feasibility of using GRPI routinely	Structured interviews
			Fidelity	GPRI implemented as intended	Clinician	Use by clinician (Y/N) and extent of use	Appt. checklist, audio recording
			Implementation cost	Health resource use	Service	Cost to service ($AUD)	Health economic data
			Penetration	Integration of GPRI in genetic counselling appointments	Clinician	No. of clinicians who use GPRI	Appt. checklist, audio recording
			Sustainability	Intention to routinely use GPRI	Clinician	Sustainability of using GPRI routinely	Structured interviews

The effectiveness of the GPRI will be tested using a superiority randomised control trial (RCT) to assess outcomes of using the GPRI during PFCC appointments and will be guided by the Framework for Outcomes of Clinical commUnication Services for Genetic Counselling (FOCUS-GC) [22]. This trial uses a parallel group design where participants will be randomised to the control group (no GPRI) or the intervention group (GPRI). Patients in the control group will receive usual care consisting of the current standard of genetic counselling where the GPRI is not used. Implementation of the GPRI will be concurrently assessed guided by Proctor’s Implementation Outcomes Framework [23]. Given the acceptability and feasibility were assessed during the pilot [20], the remaining outcomes, cost-effectiveness, adoption, feasibility of routine use, fidelity, penetration, and sustainability, will be assessed during this hybrid trial from the perspective of clinicians and the healthcare service.

#### Intervention

The GPRI is a 20-item instrument demonstrated to have high reliability (Cronbach’s alpha at 0.81), construct validity that highly correlates with other relevant psychometric scales, and a predictive value of 0.78 [19]. This tool consists of three subscales: (1) personal and family history of inherited condition; (2) perceived impact and personal adjustment to genetic testing; and (3) history of mental health concerns [19]. Nineteen of the items are summed to determine a GPRI score (range 10–100) and the scale has a clinically meaningful cutoff score of 50, which identifies 84% of patients who are at increased risk of distress after they receive their genetic test results [19]. The remaining item is used to indicate whether further counselling is needed after the genetics appointment.

Participants who are randomised into the intervention group will be emailed a link to the GPRI hosted on REDCap 3 days prior to both PFCC appointments and participants will complete the GPRI online prior to this appointment. The GPRI score is calculated using the REDCap auto-scoring capability and the completed measure and score is emailed to the relevant clinician. Given this is a low-risk intervention and the intervention involves completion of a validated screening tool, there will be no modification to the intervention. Participants in the intervention group who have not completed the GPRI within 24 h of their appointment are sent a reminder SMS that includes the URL to the online GPRI. Those who subsequently do not complete the GPRI receive usual care of standard genetic counselling and will continue in the trial for all follow-up assessments scheduled.

#### Primary outcome

The primary outcome of patient empowerment, as measured by the Genetic Counselling Outcome Scale (GCOS-24), is measured from baseline to the primary timepoint of 2 weeks after the second PFCC appointment. The GCOS-24 is validated to measure change in patient empowerment from pre- to post-genetic counselling and consists of 24 items each with a 7-point Likert scale response option scored from 1 “strongly disagree” to 7 “strongly agree” [24]. Negatively worded responses options are transformed so that all increases in scores represent an increase in empowerment and the total score is calculated by determining the sum of the scores [24].

#### Secondary outcomes

The effectiveness of using the GPRI, relative to usual care, will be further explored through secondary outcomes including change in GCOS-24 score from baseline to 2 weeks after the first PFCC appointment and proportion of respondents who meet the Minimum Clinically Important Difference (MCID) whose GCOS-24 score changes ≥10 points from baseline to 2 weeks after the second PFCC appointment [25]. Other secondary outcomes include patient satisfaction at 2 weeks after first and second PFCC appointments, patient adaptation 2 weeks and 6 months after the second PFCC appointment, and impact of genetic test result 2 weeks after the second PFCC appointment. Uptake of cancer risk management strategies and communication of genetic information to at-risk family members will be assessed at 6 months after the second PFCC appointment. The proportion of psychosocial needs identified by clinicians during the PFCC appointments, support offered after the PFCC appointments, and duration of the PFCC appointments will be established. Implementation outcomes including cost-effectiveness, adoption (uptake of the GPRI by clinicians during appointments), fidelity (use of GPRI and extent of use by clinicians during appointments), penetration (number of clinicians who use the GPRI during appointments), and clinicians’ perceptions of the feasibility and sustainability of using the GPRI routinely will also be assessed.

### Participants

#### Patients

This study will invite patients who are at increased risk of an inherited predisposition to cancer and are attending the PFCC for genetic testing. Patients attending their first appointment and who are likely to be offered genetic testing will be eligible. Involvement in this research is voluntary.

##### Eligibility

Patients will be considered eligible once their intake process is complete and their PFCC appointment has been booked. Inclusion in the study will be assessed by the research team, with input from the clinical team, based on the following criteria:

- Aged 18 years or older;
- Literate in English;
- Reasonable internet access and capacity to complete computer-based surveys; and
- Likely to be offered a genetic test during their first appointment.

Patients will be excluded if they have a known cognitive impairment or are eligible for the PRiMo study (using Polygenic Risk Modification to improve breast cancer prevention; HREC no. HREC/64060/PMCC; ANZCTR: ACTRN12621000009819). Patients will become ineligible to participate if they do not consent to genetic testing during their first appointment.

#### Clinicians

Potential participating clinicians must be employed by the PFCC at the Peter MacCallum Cancer Centre or the Royal Melbourne Hospital and involved in providing genetic counselling and testing to patients. Potential participating clinicians include associate and certified genetic counsellors, clinical geneticists, clinical genetic fellows, medical oncologists, and gastroenterologists.

### Recruitment

#### Patients

Recruitment will occur alongside standard PFCC clinical processes from May 2022 to December 2024, inclusive. Recruitment lists will be generated by reviewing the schedule for upcoming PFCC appointments and screening against the eligibility criteria. Eligible patients’ details including an email address will be entered into the study database hosted on REDCap at the Peter MacCallum Cancer Centre [26, 27]. The survey distribution tools in REDCap will be used to email study invitations. It is estimated based on the response rate of a similar genetic counselling intervention study that approximately 50% of invited patients will agree to participate [18]. Therefore, an estimated 702 potential participants will be invited to participate to achieve the requisite sample size.

The study database will house the Participant Information Statement, an e-consent framework, the GPRI, and all data collection instruments.

##### Randomisation

Randomisation of eligible patients to the intervention or control group will be conducted using randomly permuted blocks of varying sizes in a 1:1 ratio, stratified by sex and cancer status (affected or unaffected by cancer). The randomisation list will be computer-generated by an independent statistician and carried out centrally to ensure concealment. The study statistician will remain blinded until the database is cleaned, locked, and ready for unblinding. The statistical analysis plan will be written and published on our centre’s website while blind to group allocation. Blinding of patient and clinicians to the allocation status will not be possible because this is an open-label trial design.

#### Clinicians

All eligible PFCC staff will be invited to a short (30-min) study education session which will include training on how to use the GPRI. To participate in the study, clinicians agree to use the GPRI in appointments according to their clinical discretion with intervention group patients, and for all patients enrolled in the trial, audio-record the appointments and complete the post-appointment clinician checklist. Participating clinicians will also be invited to take part in a structured interview with a member of the research team.

### Data collection

#### Patient-administered measures

**Fig. 1:**
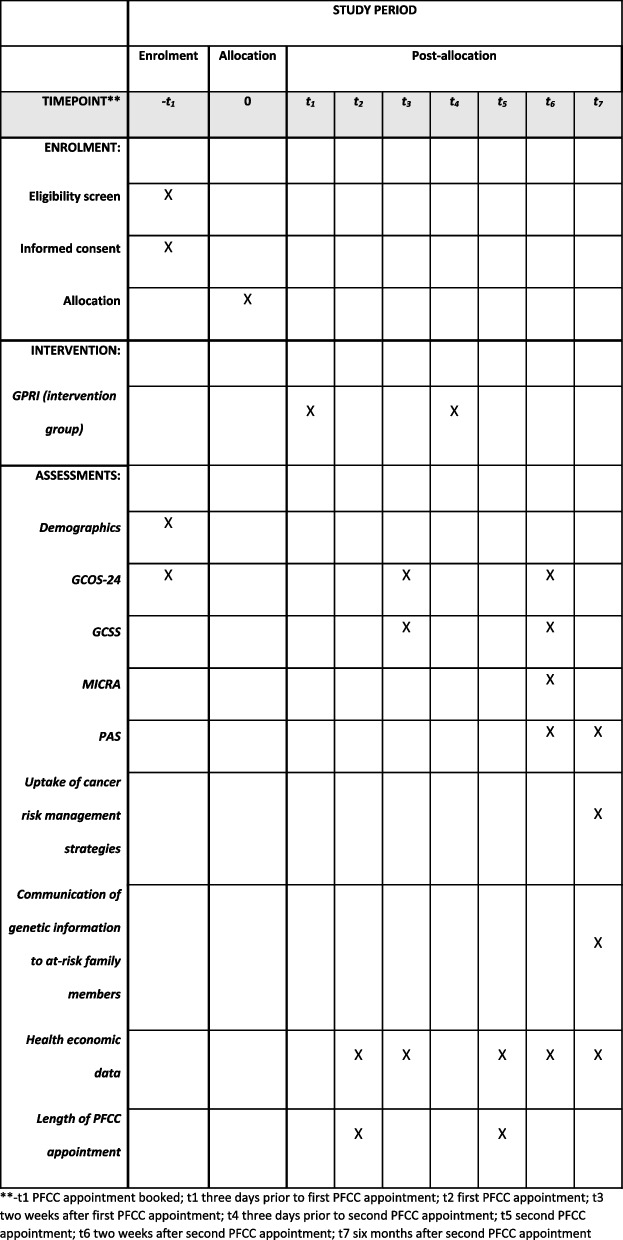
Schedule of enrolment, interventions, and assessments for the POETIC trial. **−t1 PFCC appointment booked; t1 3 days prior to first PFCC appointment; t2 first PFCC appointment; t3 2 weeks after first PFCC appointment; t4 3 days prior to second PFCC appointment; t5 second PFCC appointment; t6 2 weeks after second PFCC appointment; t7 6 months after second PFCC appointment

Participants will complete four questionnaires during the intervention phase (Fig. 1). After consenting, participants will complete the baseline questionnaire consisting of demographics and the GCOS-24. Two weeks after the first PFCC appointment, participants complete the second questionnaire including the GCOS-24 and the Genetic Counselling Satisfaction Scale (GCSS) [28]. Two weeks after the second PFCC appointment, participants complete the third questionnaire including the GCOS-24, the GCSS, the Multidimensional Impact of Cancer Risk Assessment (MICRA) [29], and the Psychological Adaptation Scale (PAS) [30]. Six months after the second PFCC appointment, participants complete the fourth questionnaire including the PAS, and purpose-designed items examining uptake of cancer risk management strategies and communication of genetic information to at-risk family members. For completion of each questionnaire, an email containing the relevant URL will be sent using the REDCAP automated survey invitation function that includes up to two reminders sent weekly to non-responders.

#### Clinical data

Clinical data (e.g., genetic testing results, the number and type of referrals for psychosocial support, letters written for at-risk family members, referrals for risk management) will be collected at various time points during the study from Epic, an electronic health records system, used at the Peter MacCallum Cancer Centre and the Royal Melbourne Hospital.

#### Clinician data

##### Clinician checklist

After each appointment, clinicians will be asked to complete a checklist that is comprised of items that mirror the GPRI domains. Clinicians will complete this electronic case report form (eCRF) to report whether each psychosocial need was discussed (yes/no) or not applicable.

##### Structured interviews

Clinicians will be asked to complete a structured interview during the post-intervention phase of the study about their experiences of using the GPRI and the feasibility and sustainability of routine use after the conclusion of the trial. Interviews will be audio-recorded with verbal consent from the clinician at the beginning of the interview. The interviews will follow a structured guide comprising two sections: (1) demographics and (2) feasibility and sustainability of using the GRPI in routine practice. The demographics section will include profession, number of years practicing, and estimated number of times using the GPRI. Section 2 will include questions to explore: the clinician’s experiences of using the GPRI during appointments and whether they believe the GPRI to be a feasible and sustainable tool to use as part of routine care.

#### Healthcare resource use

A cost analysis comparing the intervention and control groups will be conducted from both a societal and public healthcare system perspective, using a bottom-up micro-costing approach. Clinicians will be asked to enter data pertaining to the time spent (in minutes) on administrative and clinical tasks for patients involved in the study. Clinicians will be asked to enter this data for 50 patients enrolled during the first 6–12 months of the intervention period and for 50 patients in the final 6–12 months. This will prevent over-burdening clinicians with data entry tasks and will allow analysis of the impact of gain of experience in using the GPRI.

#### Appointment data

Clinicians will be supplied with Dictaphones and asked to audio-record each appointment. Consent will be implied for clinicians through the opt in process and signing the delegation log and obtained from participants as part of their consent to take part in the overall study. At the end of each clinic, a member of the research team will collect the Dictaphones and transfer the audio recording onto the PFCC network drive. A member of the research team will listen to each appointment audio recording and, using a checklist, will record the frequency that psychosocial concerns included in the GPRI were discussed and whether psychosocial supports were offered. During the appointments where the GPRI was available, the audio recording will be reviewed to examine the adoption by clinicians of the GPRI during appointment, whether the GPRI was used as intended to assess fidelity, and the integration of the GPRI results in the appointment to examine penetration. The member of the research team involved in this aspect of study will not be associated with the clinician team to preserve clinician anonymity. The audio recordings will also be used to determine the length of each appointment.

#### Health economic data

Clinical data, patient-reported data, appointment data, and clinician data will be collated and used for the health economic analysis. Only costs directly related to the study intervention/genetic condition will be considered. The cost per patient will be established by (1) identification of relevant clinical pathways in consultation with study investigators, (2) estimate resource measurement for each pathway, (3) estimation of the unit cost for each resource, and lastly (4) application of the cost of resources to each clinical pathway. The cost of staff time will be based on time spent and the relevant gross salary award adjusted for on-costs (leave, leave-loading). Productivity costs (work absences, lost income) and out-of-pocket and indirect patient costs will be estimated using patient data collected through the clinical questionnaires.

## Data analysis

### Sample size

A total sample size of 246 participants, 123 per group, is required for 90% power to demonstrate that GPRI superior to usual care with a two-sided 5% significance level. This sample size is based on the following assumptions: a clinically meaningful absolute treatment difference in mean change in GCOS-24 scores from baseline to 2 weeks after the second PFCC appointment of 10 units in favour of GPRI (increase) [25], standard deviation (SD) of 20 units equal in each group, and at each time point (baseline, 2 weeks after first PFCC appointment, and 2 weeks after second PFCC appointment) [25], and conservatively no correlation between baseline and repeated measures of GCOS-24 scores. Attrition is estimated to be similar to another genetic counselling intervention study where approximately 30% participant attrition was observed [31].

### Statistical analysis

The biostatistician will devise a formal detailed statistical analysis plan for the study prior to unblinding. The analysis set will consist of all participants who were randomised, with all participants reported and analysed according to their randomised study arm. There is no interim-analysis planned due to the low risk of harm to participants presented by this trial.

#### Primary outcome

Primary outcome GCOS-24 scores will be analysed using a constrained longitudinal data analysis (cLDA) [32] model assuming a common mean across groups at baseline and a different mean for each group at each follow-up time point. The response will consist of all GCOS-24 scores (scores at baseline, and at 2 weeks after the first PFCC appointment and 2 weeks after the second PFCC appointment) and the model will include a random intercept for clinician and an unstructured variance-covariance matrix for the individual’s repeated measurements, factors representing treatment, time (categorical), and treatment-by-time interaction, and the stratification factors sex and cancer status at baseline, and will be further adjusted for cancer type. The mean change in GCOS-24 scores from baseline to each follow-up time point between the intervention group compared to the control group will be obtained. The primary hypothesis will be evaluated by obtaining the estimated difference between intervention and control groups in mean change in GCOS-24 score from baseline to 2 weeks after the second PFCC appointment, a two-sided 95% confidence interval and a *p*-value. This model provides valid inference in the presence of missing data if the data are missing at random. An analysis will be conducted using the delta-adjustment method under the pattern-mixture modelling framework in the context of multiple imputation to assess sensitivity to missingness not at random.

#### Secondary outcomes

In addition to the primary analysis of the GCOS-24 score, responders (defined as change from baseline to 2 weeks after second PFCC appointment in GCOS score ≥ 10) will be analysed using a logistic regression model using generalized estimating equations (GEEs) with an exchangeable correlation structure and robust standard errors for clustering by clinicians [33]. Continuous secondary outcomes with repeated measures—satisfaction with genetic counselling and adaptation to genetic risk—will be analysed using a linear mixed-effects model. The model will include a random intercept for clinician and an unstructured variance-covariance matrix for the individual’s repeated measurements and factors representing treatment, time (categorical), and treatment-by-time interaction. Other secondary outcomes, impact of genetic test result, and duration of PFCC appointments will be analysed using linear regression, total number of psychosocial needs identified in each appointment will be analysed using Poisson regression, and support offered to patients after appointments will be analysed using logistic regression. All these models will be fitted using GEEs with an exchangeable correlation structure and robust standard errors for clustering by clinicians and will be adjusted for the stratification factors sex and cancer status, with further adjustment for cancer type. These models provide valid inference in the presence of missing data if the data are missing completely at random (MCAR). Sensitivity analyses will be conducted using multiple imputation to explore the impact of any deviations from MCAR on the results.

Subgroup analyses for the comparison of the primary outcome, patient empowerment measured using the GCOS-24 score, for participants in the intervention group compared to the control group will be conducted by the stratification factors sex, cancer status (affected vs unaffected), and additionally by type of clinician (genetic counsellors vs doctors), including subgroup and an interaction term between treatment and subgroup in the model. All analyses of secondary outcomes and subgroups are exploratory and therefore require no adjustment for multiple testing. All statistical analyses will be conducted using Stata statistical software [34].

#### Health economic analysis

Resources will be itemised and valued in 2020 Australian Dollars (AUD). A cost analysis comparing control and intervention groups will be conducted from both a societal and public healthcare system perspective, using a bottom-up micro-costing approach. Costs will be compared using mean estimates, or through nonparametric bootstrap in the event of skewed data.

### Data management, retention, and storage

All electronic data will be stored in REDCap. Data exported from REDCap during analyses will be stored in a secure folder on the internal network drive. All electronic files and the final cleaned dataset will only be accessible to the investigator team. Audio recordings of the appointments and the structured interviews will be deleted from the Dictaphone after transfer to the network drive.

There is no data monitoring committee appointed for this trial due to the minimal risk presented by this research.

All files and data relating to this project will be kept for a minimum of 5ive years in accordance with the guidelines stipulated by the National Health & Medical Research Council in the Australian Code for the Responsible Conduct of Research. After this time, electronic data will be permanently deleted.

### Ethical considerations and risk mitigation

The study will be conducted according to the National Health and Medical Research Council (NHMRC) National Statement on Ethical Conduct in Human Research 2007 (and updates), and the World Medical Association Declaration of Helsinki 2013. Any modifications to the protocol that change the study design or conduct, or significant administrative changes, will require a formal amendment that will be reviewed and approved by the sponsor, the Peter MacCallum Cancer Centre, and the Peter MacCallum HREC prior to implementation. These changes will also be reflected as updates to the trial registration at the Australian New Zealand Clinical Trials Registry.

#### Management of distress

This study is not expected to detract from the usual standard of care for patients, as the GPRI has been validated to detect psychosocial needs in the clinical genetics setting and enhance communication between staff and patients. Any patients who experience distress during an appointment are counselled about the issues causing their distress during the appointment, relevant supports are identified, and referrals are made to appropriate psychological support services, if required. If a staff member identifies a participant is distressed based on their GPRI results, they will follow these standard clinical processes.

This study is not expected to cause any distress to clinical staff who participate or to members of the research team. However, if a staff member is distressed due to the completion of the staff questionnaire, they will be provided with support by the Director of the PFCC.

### Dissemination of findings

The progress of the study and the study findings at completion will be communicated locally and internationally. Results will also be published in peer-reviewed journals and disseminated to genetic and oncology health professionals and researchers at national and international conferences. Authorship on any publications arising from this trial will be determined by guidelines published by the NHMRC that support the Australian Code for the Responsible Conduct of Research and there are no plans to use any professional writers for this process.

## Discussion

The POETIC trial takes a pragmatic approach, complementing the hybrid design, by deploying the GPRI as an intervention in the routine clinical practice of a Familial Cancer Centre staffed by a multidisciplinary team of genetics and oncology clinicians. Following this pragmatism, the sample population will not be highly selected based on narrow eligibility criteria, and instead most patients referred to the PFCC will be eligible to be invited to the study. All clinicians comprising genetic counsellors, medical speciality trainees, and medical practitioners will have opportunity to use the GPRI and have their practice evaluated as part of this trial. While a consequence of this pragmatic design means that a larger sample size is needed to evaluate the primary outcome, a corollary may be that the effectiveness and implementation evidence generated from this real-world health service setting will optimise the relevance of the trial to, and enhance operationalisation of the screening tool in, routine practice.

Germline genetic testing has tangible clinical health outcomes in terms of diagnosis and risk prediction, but also has the potential to impact psychosocial health of individuals and families. However, the provision of genetic counselling, with a beneficial outcome defined by patients as empowerment, is an opportunity to facilitate informed decision-making and psychological adaptation in relation to a genetic condition [9]. In the demanding clinical environment of clinical genetics services, use of the GPRI screening tool may create capacity to personalise the practice of genetic counselling though identifying and focusing on the aspects of most concern for each client. In this POETIC trial, the effectiveness of the GPRI in the familial cancer setting will be measured primarily in terms of the widely accepted genetic counselling outcome of empowerment using the rigorously validated GCOS-24 scale [24]. Should the GPRI be demonstrated to be effective in this clinical genetics setting, it could then be implemented and trialled as an intervention in other health service settings, such as mainstreaming models, where genetic testing is increasingly being facilitated by non-genetics specialists. This would provide consistent evidence across settings of whether the GPRI can standardise and streamline genetic counselling, irrespective of the provider, to safeguard patient outcomes after genetic testing.

## Trail status

Protocol V2 08.11.2021; recruitment commenced on May 19, 2022. It is estimated that recruitment will be completed by December 31, 2024.

### Supplementary Information

**Additional file 1.**

**Additional file 2.**

## Data Availability

Not applicable.
