# Supplementary material for: Personalising genetic counselling (POETIC) trial: Protocol for a hybrid type II effectiveness-implementation randomised clinical trial of a patient screening tool to improve patient empowerment after cancer genetic counselling
Source: Trials. 2023 Nov 8;24:712. doi: 10.1186/s13063-023-07723-0 (PMC10631179; doi:10.1186/s13063-023-07723-0)
Supplement: Supplementary file 2 — Additional file 2. [file 13063_2023_7723_MOESM2_ESM.docx]

Appendix A

| **HREC Project Number** | HREC/78093/PMCC-2021 | | |
| --- | --- | --- | --- |
| **Research Project Title** | The PersOnalising gEneTic Counselling Trial:  Testing the implementation and effectiveness of an intervention to personalise genetic counselling | | |
| **Short title** | POETIC Trial | | |
| **Principal Researcher** | Dr Laura Forrest Senior Research Fellow Parkville Familial Cancer Centre Peter MacCallum Cancer Centre | | |
| **Version Number** | 1.3 | **Version Date:** | 26.11.21 |

Dear [Name],

We are inviting you to take part in our research project called the **‘POETIC Trial’.** You are being invited because you have a genetics appointment coming up at the Parkville Familial Cancer Centre.

This research is testing whether using a **Patient Screening Tool** in a genetics appointment changes the outcomes for patients after their appointment. Patient screening tools are commonly used in many healthcare settings. They help clinicians quickly identify patient needs. We need more research to understand if patient screening tools are helpful in genetics appointments.

Some people who take part will be asked to complete this screening too. This screening tool will be used by your genetic clinician during your genetics appointment, to try and make sure any questions and/or concerns you may have will be answered.

**Participant Information Statement**

This **Participant Information Statement** tells you about the research project and explains what you will be asked to do as part of the project. We are hoping that knowing what is involved will help you to decide if you would like to take part in the research.

**Before you agree to be part of the POETIC Trial:**

1. Please read this information carefully.
2. Please ask questions about anything that you don’t understand or want to know more about. Before deciding whether or not to take part, you might also want to talk about it with a relative, friend or your local doctor.
3. If you decide you want to take part in the research project, you will be asked to electronically sign the Consent Form.
4. By signing the Consent Form you are telling us that you:

• Understand what you have read

• Consent to taking part in the research project

• Consent to participating in the study procedures that are described below

• Consent to the use of your personal and health information as described.

For you reference, you will be sent an email with this **Participant Information Sheet** and **Consent Form** after you have read it.

**What does participation involve?**

You will be participating in a randomised controlled research project. We do not know whether using a Patient Screening Tool in a genetics appointment is better than current standard practice. To find out, we need to compare using this tool to the current practice. To do this, we put participants into two groups:

**Group 1**: will not complete the patient screening tool and will receive current standard practice.

**Group 2:** will complete the patient screening tool prior to their genetics appointments for use during the appointment.

Participants will be put into group 1 or group 2 by chance (randomly). Participants will be allocated to a group after they have completed the first research survey for this study.

At the end of the trial, we will compare the results from the two groups.

**What will I be asked to do?**

As part of this study, **all** participants will be asked to:

1. **Complete an online survey before your first genetics appointment:**  This survey has questions about your background, like your age, sex, education, and about your family. It also has questions about your upcoming genetics appointment. It will take about **10 minutes** to complete. To take part in this study, you need to finish this survey before your genetics appointment.
2. **Allow us to audio-record your genetics appointments:** We will use this audio recording to see if the questions and/or concerns you have in your appointment are answered.
3. **Complete short online surveys about your experience of having genetics appointments:** You will be invited to complete **3 surveys** over the study period. The surveys will be emailed to you:

- **2 weeks** after your genetics appointment,
- **2 weeks** after receiving your genetic test results, and
- **6 months** after receiving your genetic test results

Each survey will take **15-20 minutes** to finish.

1. **Please allow us to access your clinical information:** Patients at the Parkville Familial Cancer Centre have their own electronic medical record (EMR). During the study, we will access your EMR to collect information about your genetics appointment and your clinical outcomes. This may include:

- Genetic test results
- Details of screening tests, procedures, surgeries and/or use of medications
- Clinical advice relating to cancer treatment, prevention or risk assessment

Participants who are put in group 2 will be asked to please complete the Patient Screening Tool within **3 days** of their genetics appointment. A link to the tool will be emailed to participants in this group before their appointment.

Participants who are **selected to complete** the Patient Screening Tool will be asked to answer **19 questions** that form the screening tool, about their thoughts and feelings about their upcoming genetics appointment.

**Are there any benefits to taking part?**

The aim of the research project is to help us to improve our clinical genetics service at the Parkville Familial Cancer Centre. Your feedback and commitment to participating in this project may benefit others in the future.

**Are there any risks to taking part?**

We have tried to be sensitive to your feelings and experiences by making sure that the questions in the surveys do not upset you. However, if you are worried or concerned about any of the questions, you can contact us at any time during the project.

**Do I have to take part?**

Participation in this project is voluntary. This means that it is your choice and you do not have to take part if you do not want to.

Your decision whether to take part or not to take part, or to take part and then withdraw, will not affect your current or future medical care in any way. You will be able to attend your appointment at the Parkville Familial Cancer Centre as planned and receive the current standard of care.

If you decide to participate and later change your mind, you can withdraw from the study and ask that any information you have provided is destroyed. If you would like to withdraw, please fill out a **Withdrawal of Participation Form** and email it to [poetictrial@petermac.org](mailto:poetictrial@petermac.org). You can find this form in the email sent to you after you read the **Participant Information Sheet.**

**What will happen to the information about me?**

By signing the Consent Form you consent to the research team collecting and using personal information about you for the research project.

Any information collected in connection with this research project that can identify you will remain confidential. It will only be disclosed with your permission, except as required by law.

Information about you will be collected from your EMR. By signing the Consent Form you agree to the research team accessing information in your EMR if it is relevant to your participation in this research project. A copy of your signed Consent Form will be added to your EMR, and your clinician will know that you are taking part in the project.

Information you provide to the research team or give us permission to collect, will be stored electronically in a secure database or restricted electronic folders, housed on secure servers at the Peter MacCallum Cancer Centre. Only those persons authorised will have access to the information.

If you are selected to complete the screening questionnaire, your answers will be shared with your genetics clinician. All other survey answers will not be shared with your clinician. A member of the research team will listen to each appointment audio recording and use a checklist to record whether topics covered in the screening tool were raised in your appointment.

When your information is analysed, all identifiers (e.g. name and personal details) will be removed and replaced with a code. It will be possible to re-identify the information as yours by using the code.

Results from this research project will be published in scientific or medical journals and presented at scientific meetings and potentially in the wider media. For these reports no identifying information will be included, and care will be taken to ensure that it is not possible to identify you from public reports, except where you have given your written expressed permission.

In accordance with the institutional policy of Peter MacCallum Cancer Centre where this research project is being conducted, your study records and data (including appointment audio recordings) will be retained for a minimum of 15 years from the date of completion of all research activities.

After the completion of the study and at the end of the retention period, your confidential information that has been stored electronically will be destroyed securely using a specialized ‘shredding’ software and any hard copy documents will be shredded. If you would like to withdraw from the study, you can ask for your information to be destroyed in the same way.

**Who do I contact for more information?**

If you have any questions, concerns or would like further information about this project, please email or call:

**Dr Laura Forrest at** [**laura.forrest@petermac.org**](mailto:laura.forrest@petermac.org) **or (03) 8559 6191**.

If you have any concerns and/or complaints about the project, the way it is being conducted or your rights as a research participant, and would like to speak to someone independent of the project, please contact:

**Ethics Coordinator, Peter MacCallum Human Research Ethics Committee on telephone:**

**(03) 8559 7540.**

*This research project has been approved by the Peter MacCallum Cancer Centre Human Research Ethics Committee and will be carried out in line with the National Statement on Ethical Conduct in Human Research (2007) – including all updates.*

**Consent to participate**

If you agree to take part in this project, please tap the “I agree” button below.

This will take you to the next screen where the survey will start.

If you do not agree to take part, please tap the “I do not agree” button.

I agree

I do not agree

First name:

Last name:

Date of Birth:

Signature: _________________________

**Form for Withdrawal of Participation**

| **HREC Project Number** | HREC/78093/PMCC-2021 |
| --- | --- |
| **Research Project Title** | The PersOnalising gEneTic Counselling Trial:  Testing the implementation and effectiveness of an intervention to personalise genetic counselling |
| **Short title** | POETIC Trial |
| **Principal Researcher** | Dr Laura Forrest Senior Research Fellow Parkville Familial Cancer Centre Peter MacCallum Cancer Centre |

I wish to withdraw my participation in the above research project

I understand that withdrawing participation does not affect my care or relationship with the Parkville Familial Cancer Centre

*Optional*

I would like any information I have already provided to the study to be destroyed immediately

| Name: | |
| --- | --- |
| Signature: | Date: |
